# Adaptive Stress Response in Segmental Progeria Resembles Long-Lived Dwarfism and Calorie Restriction in Mice

**DOI:** 10.1371/journal.pgen.0020192

**Published:** 2006-12-15

**Authors:** Marieke van de Ven, Jaan-Olle Andressoo, Valerie B Holcomb, Marieke von Lindern, Willeke M. C Jong, Chris I. De Zeeuw, Yousin Suh, Paul Hasty, Jan H. J Hoeijmakers, Gijsbertus T. J van der Horst, James R Mitchell

**Affiliations:** 1 Medical Genetics Center, Department of Cell Biology and Genetics, Center of Biomedical Genetics, Erasmus Medical Center, Rotterdam, The Netherlands; 2 Department of Molecular Medicine, University of Texas/Institute of Biotechnology, San Antonio, Texas, United States of America; 3 Department of Hematology, Erasmus MC, Rotterdam, The Netherlands; 4 Department of Neuroscience, Erasmus MC, Rotterdam, The Netherlands; Stanford University School of Medicine, United States of America

## Abstract

How congenital defects causing genome instability can result in the pleiotropic symptoms reminiscent of aging but in a segmental and accelerated fashion remains largely unknown. Most segmental progerias are associated with accelerated fibroblast senescence, suggesting that cellular senescence is a likely contributing mechanism. Contrary to expectations, neither accelerated senescence nor acute oxidative stress hypersensitivity was detected in primary fibroblast or erythroblast cultures from multiple progeroid mouse models for defects in the nucleotide excision DNA repair pathway, which share premature aging features including postnatal growth retardation, cerebellar ataxia, and death before weaning. Instead, we report a prominent phenotypic overlap with long-lived dwarfism and calorie restriction during postnatal development (2 wk of age), including reduced size, reduced body temperature, hypoglycemia, and perturbation of the growth hormone/insulin-like growth factor 1 neuroendocrine axis. These symptoms were also present at 2 wk of age in a novel progeroid nucleotide excision repair-deficient mouse model (XPD^G602D/R722W^/XPA^−/−^) that survived weaning with high penetrance. However, despite persistent cachectic dwarfism, blood glucose and serum insulin-like growth factor 1 levels returned to normal by 10 wk, with hypoglycemia reappearing near premature death at 5 mo of age. These data strongly suggest changes in energy metabolism as part of an adaptive response during the stressful period of postnatal growth. Interestingly, a similar perturbation of the postnatal growth axis was not detected in another progeroid mouse model, the double-strand DNA break repair deficient *Ku80* ^−/−^ mouse. Specific (but not all) types of genome instability may thus engage a conserved response to stress that evolved to cope with environmental pressures such as food shortage.

## Synopsis

Oxidative damage to cellular components, including fats, proteins, and DNA, is an inevitable consequence of cellular energy use and may underlie both normal and pathological aging. Calorie restriction delays the aging process and extends lifespan in a number of lower organisms including rodents. Inborn defects in the postnatal growth axis resulting in dwarfism can also extend lifespan. Both may function via overlapping pathways impacting on energy metabolism. Here, we report a novel DNA repair-deficient mouse model with symptoms of the related premature aging disorders Cockayne syndrome and trichothiodystrophy, namely reduced fat deposits, neurological dysfunction, failure to thrive, and reduced lifespan. Surprisingly, we also observed traits usually associated with extended longevity as found in calorie restriction and dwarfism, including reduced blood sugar and reduced insulin-like growth factor-1. These characteristics were present at 2 wk of age, that is, during the period of rapid postnatal development, but returned to normal by sexual maturation at 10 wk. Furthermore, they were absent altogether in another premature aging mouse model with a distinct DNA repair defect. Specific types of unrepaired DNA damage may thus elicit a preservative organismal response affecting energy metabolism that is similar to the one that evolved to cope with the stress of food shortage.

## Introduction

Congenital defects in genome stability-promoting mechanisms such as DNA repair often lead to elevated cancer predisposition and/or the accelerated appearance of some but not all characteristics (“segments”) observed in natural aging [1]. Among these so-called segmental progerias, different defects lead to the acceleration or exaggeration of different segments of aging. Typical examples include Werner syndrome, in which diabetes, osteoporosis, and increased cancer incidence are observed, and Cockayne syndrome, characterized by neurological dysfunction and cachectic dwarfism. Although the causative genetic defects of many progerias are known, the molecular mechanisms underlying pleiotropic disease symptoms remain elusive and their relevance to normal aging controversial [2]. However, because cultured fibroblasts from almost all reported mouse models of segmental progeria (as well as many of the corresponding human progerias) exhibit limited proliferative capacity [3], cell-based mechanisms including premature senescence and enhanced apoptosis have emerged as strong candidates underlying pleiotropic disease symptoms [4,5].

The evolutionarily conserved nucleotide excision repair (NER) pathway removes helix-distorting DNA damage (such as UV lesions) in a complex “cut and patch” mechanism. Inborn NER defects can lead to the skin cancer syndrome xeroderma pigmentosum (XP) or the segmental progerias Cockayne syndrome (CS) and trichothiodystrophy (TTD) [6]. An increase in the mutation rate caused by defects in one of at least seven different NER proteins (XPA–XPG) explains the cancer predisposition in XP [7]; each of these proteins participates in lesion removal anywhere in the genome (global genome NER). In contrast, how alterations in any of five NER-associated proteins (XPB, XPD, XPG, CSA, and CSB) can cause progeria remains largely obscure. Current genetic evidence points to a link with defective transcription-coupled repair [8–11], a subpathway of NER that specifically removes lesions on the transcribed strand of active genes that interfere with transcription, thus promoting cellular survival from DNA damage [12,13].

The *XPD* gene, encoding a helicase subunit of the transcription/NER-associated transcription factor II H complex, is unique amongst NER genes in that different point mutations are associated with cancer (XP), progeria (TTD), or a combination (XP combined with Cockayne syndrome [XPCS] or trichothiodystrophy [XPTTD]) [8,14,15]. Although the hallmark feature of TTD, brittle hair, is not observed in CS or XPCS, the neurodevelopmental features of the diseases are similar [16,17]. In most cases these features include (i) normal in utero development followed by postnatal growth failure in the first year (including reduced postnatal brain growth [18]); (ii) progressive gait abnormalities caused by a combination of cerebellar ataxia, joint contractures, spasticity, and frequent kyphosis; (iii) sensorineural hearing loss; (iv) progressive loss of subcutaneous fat (cachexia) yielding an “aged” appearance; and (v) the conspicuous lack of (skin) cancer predisposition despite sun sensitivity due to a cellular UV repair defect [16,17].

We mimicked point mutations found in human TTD *(Xpd^TTD^)* and XPCS *(Xpd^XPCS^)* patients in the mouse and reported phenotypic overlap between homozygous mutant animals and the corresponding human syndromes [9,10]. Crossing mutant *Xpd* animals allowed us to address the effects of compound heterozygosity, or the presence of two different mutant alleles of the same gene, on disease symptoms. Compound heterozygosity between *Xpd^TTD^* and *Xpd^XPCS^* alleles surprisingly ameliorated developmental delay and age-related premature cachexia and kyphosis associated with each homozygous mutant mouse model as well as the cutaneous abnormalities specific to the TTD model [19].

Exacerbation of the repair defect by *Xpa* ablation, on the other hand, results in a much more severe phenotype including dramatic postnatal growth retardation, progressive cerebellar ataxia, lack of subcutaneous fat, and death around weaning in several of these mouse models (e.g., XPCS/XPA, TTD/XPA, and CSB/XPA) [9,20,21]. We refer to the above segmental progeroid phenotypes as “progeroid NER syndrome.” Here, we tested the ability of compound heterozygosity at the *Xpd* locus in an XPA-deficient background (*Xpd^TTD/XPCS^/Xpa^−/−^*) to ameliorate the severe symptoms of progeroid NER syndrome associated with the corresponding double homozygous (*Xpd^TTD/TTD^/Xpa^−/−^* and *Xpd^XPCS/XPCS^/Xpa^−/−^*) animals.

## Results

### XPD Compound Heterozygotes in an XPA-Deficient Background

A comparison amongst the subset of single and combined NER defects resulting in a severe segmental progeroid phenotype is presented in Table 1. Intercrossing of *Xpd^+/TTD^*/*Xpa^−/−^* and *Xpd^+/XPCS^*/*Xpa^−/−^* animals resulted in double homozygous *Xpd^TTD/TTD^*/*Xpa^−/−^* and *Xpd^XPCS/XPCS^*/*Xpa^−/−^* mutant mice (subsequently referred to as TTD/XPA and XPCS/XPA animals, respectively) as well as compound heterozygous *Xpd^XPCS/TTD^*/*Xpa^−/−^* (CH/XPA) mice. Expected at a Mendelian frequency of 25%, XPCS/XPA and TTD/XPA mice were instead observed at 15% and 5%, respectively, upon genotyping at postnatal day 9–10. CH/XPA animals were present at 20%, closer to the expected ratio of 25%. *Xpd^WT/XPCS or TTD^*/*Xpa^−/−^* (subsequently referred to as XPA) animals were used as controls in subsequent experiments, because XPA deficiency on its own has no or relatively minor effects on growth, development, or fecundity [22,23].

**Table 1:**
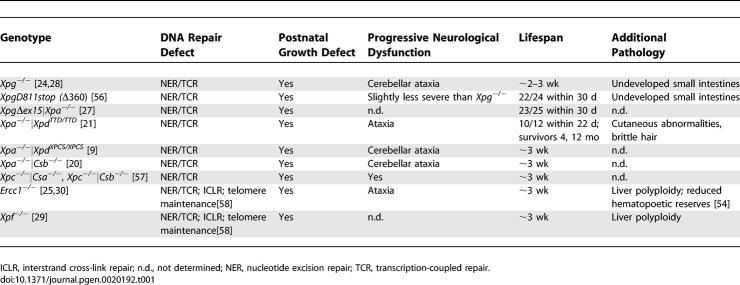
Characteristics of Progeroid NER Syndrome in Mice

**Figure 1:**
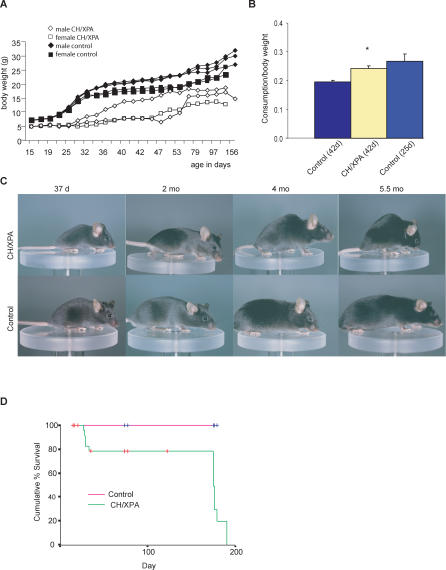
Compound Heterozygosity at the *Xpd* Locus Partially Rescues Severe Progeroid NER Syndrome (A) Growth curves of individual CH/XPA and control XPA animals from a representative litter. (B) Food intake of CH/XPA animals versus XPA littermate controls (42 d old) and 25-d-old controls expressed per gram body weight. Asterisks indicate statistical significance (*p* < 0.001) versus 42-d-old controls; error bars indicate standard error of the mean (SEM). (C) Photographs of male littermate CH/XPA and control XPA mice over 5.5 mo. (D) Kaplan-Meier survival curve of CH/XPA and littermate control XPA mice.

Although indistinguishable from control littermates after birth, indicative of normal in utero development, CH/XPA mice were visibly smaller than XPA littermates by 2 wk of age (Figure 1A and unpublished data), similar to XPCS/XPA and TTD/XPA mice [9,21]. CH/XPA mice also displayed abnormal gait and tremors (unpublished data) consistent with cerebellar ataxia as previously observed in several other progeroid NER-deficient mouse models (Table 1). However, unlike XPCS/XPA and TTD/XPA, CH/XPA mice survived the stressful weaning period with high penetrance (∼80%), reaching an average body weight about half that of littermate controls (Figure 1A).

Despite their small size, we consistently noted unusual daytime feeding behavior in CH/XPA mice, prompting an investigation into their average daily food intake. At 6 wk of age, mutant mice consumed 23% more food per gram of body weight than littermate controls (*p* = 0.001; Figure 1B). In a repeat experiment with 7-wk-old mice from a different litter, CH/XPA animals ate 12% more than littermate controls (*p* = 0.09). In both cases, food consumption per gram of body weight was similar between CH/XPA mice and younger (3- to 5-wk-old) control mice having similar weights but growing faster (Figure 1B and unpublished data). We conclude that food consumption corrected for body weight in CH/XPA mice was equal to or greater than that of littermate controls and thus does not explain their smaller size. Furthermore, at 10 wk of age, CH/XPA mice weighed on average 71% of littermate XPA controls and had a cachectic appearance. Corrected for body weight, white adipose tissue deposits were on average 48% of those of controls (perigonadal 41%, mesenteric 52%, adrenal 37%, and subcutaneous 57%; overall *p* = 0.01). Interestingly, however, interscapular brown adipose tissue deposits corrected for body weight were similar between CH/XPA mice and littermate controls at 10 wk of age. Together these data suggest that CH/XPA mice differ from control mice in either the extraction or use of energy from their food.

Between 4 and 6 mo of age, we observed a rapid and general decline in the overall condition of CH/XPA mice. Prior to sacrifice due to poor health status, mice were on average 56% of littermate weight and increasingly cachectic, with white adipose tissue corrected for body weight on average 43% of that of controls (perigonadal 43%, mesenteric 32%, adrenal 20%, and subcutaneous 63%; overall *p* = 0.02) and also reduced interscapular brown adipose tissue (62% of control, *p* = 0.01). This decline was accompanied by other progeroid features, including disturbed gait, frequent loss of balance, and kyphosis (Figure 1C; Video S1). Kaplan-Meier survival plots revealed a significant difference between CH/XPA mice and XPA littermate controls (log-rank *p* < 0.01) (Figure 1D). Mortality was observed primarily at two points, the first around weaning (approximately 1 mo of age) and the second around 5 mo of age. Mean lifespan was approximately 5 mo, which is proportional to the percent lifespan of the related human progeria, CS [16]. Thus, among the reported progeroid NER mouse models summarized in Table 1, this mutant is unique in its ability to reach adulthood with high penetrance despite ataxia and cachectic dwarfism.

### Accelerated Fibroblast Senescence Undetected in Multiple Progeroid NER Syndromes

Among NER-deficient mouse models for which cellular growth parameters have been reported, an inverse correlation appears to exist between cellular proliferative capacity in vitro and progeroid features in vivo. For example, primary mouse embryonic fibroblasts (MEFs) from *Xpg^−/−^* [24] and *Ercc1^−/−^* [25] embryos undergo premature senescence when cultured under atmospheric (∼20%) oxygen tension. XPA MEFs, on the other hand, have normal proliferative capacity [26], and the corresponding animals, despite their NER deficiency, lack profound symptoms of progeroid NER syndrome [23].

**Figure 2:**
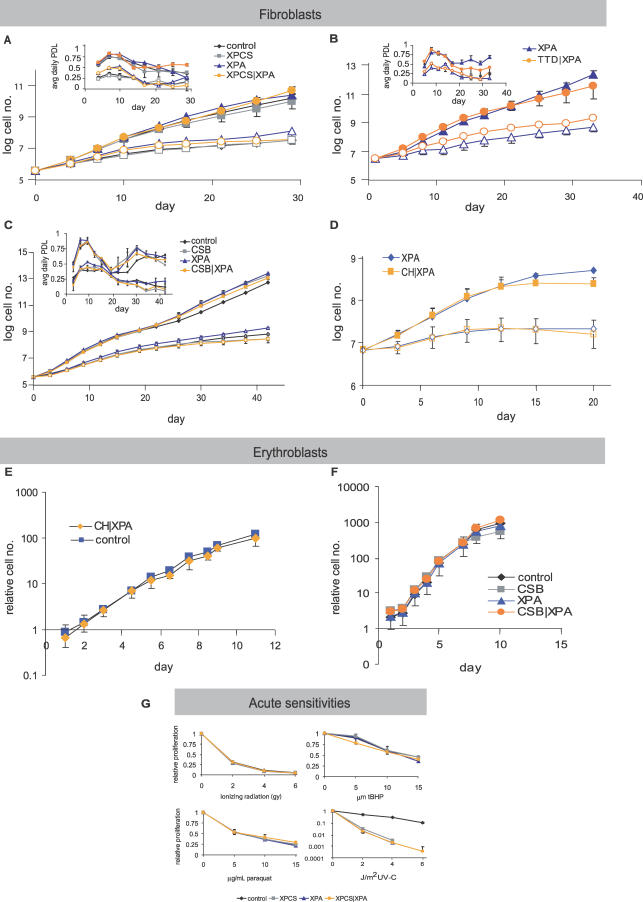
Normal Proliferation of Progeroid NER Primary Cells upon Chronic and Acute Oxidative Stress In Vitro (A–C) Serial passaging of MEFs from individual litters at 3% (filled symbols) versus 20% (open symbols) oxygen tension. Lines are compiled from the indicated number of fibroblast cultures derived from single embryos and plotted as the log of the cumulative cell number over time; error bars indicate SEM. Insets are the same data plotted to indicate the rate of growth; an average daily population doubling (PDL) of one indicates that the cells divide on average once a day. (A) Black diamonds, control (*n* = 3); blue triangles, XPA (*n* = 1); grey squares, XPCS (*n* = 2); orange circles, XPCS/XPA (*n* = 2). (B) Blue triangles, XPA (*n* = 4); orange circles, TTD/XPA (*n* = 2). (C) Black diamonds, control (*n* = 1); blue triangles, XPA (*n* = 2); grey squares, CSB (*n* = 2); orange circles, CSB/XPA (*n* = 3). (D) Serial passaging of mouse dermal fibroblasts prepared from tails of 10-wk-old animals at 3% (filled symbols) versus 20% (open symbols) oxygen tension. Lines are compiled from the indicated number of fibroblast cultures derived from single animals and plotted as the log of the cumulative cell number over time; error bars indicate SEM. (E and F) Serial passaging of erythroblasts prepared from embryonic day 12.5 embryos from a representative litter. (E) Blue squares, control (*n* = 5); orange diamonds, CH/XPA (*n* = 4). (F) Black diamonds, control (*n* = 2); blue triangles, XPA (*n* = 3); grey squares, CSB (*n* = 1); orange circles, CSB/XPA (*n* = 2). (G) Proliferative capacity upon acute treatment with paraquat, ionizing radiation, tert-butylhydroperoxide (tBHP), and UV-C as indicated is expressed as a percentage of growth of the untreated samples. Black diamonds, control (*n* = 3); blue triangles, XPA (*n* = 1); grey squares, XPCS (*n* = 2); orange circles, XPCS/XPA (*n* = 2); error bars indicate SEM.

We tested this correlation in XPD/XPA MEFs by measuring proliferation in log-phase cultures at atmospheric (20%) oxygen tension as well as low (3%) oxygen tension, in which cellular senescence is rescued in wild-type (WT) MEFs [26]. Despite the severe progeroid symptoms in XPCS/XPA and TTD/XPA mice in vivo, we found no difference in the long-term proliferative capacity of XPCS/XPA or TTD/XPA MEFs in vitro relative to control or single homozygous mutant littermate controls (Figure 2A and 2B). We also tested MEFs from another severely affected progeroid NER model, CSB/XPA (Table 1), and found no difference between control, single, or double homozygous mutant MEFs (Figure 2C). As a positive control, *Ercc1^−/−^* MEFs (*n* = 4) displayed reduced proliferative capacity versus heterozygous or WT controls (*n* = 4) at 20% oxygen tension (unpublished data) as previously reported [25].

In order to see if this unexpected lack of premature cellular senescence extended to cells prepared from adult animals or to another cell type, we tested the proliferative capacity of dermal fibroblasts prepared from the tails of 10-wk-old CH/XPA mice and erythroblasts prepared from embryonic day 13.5 fetal livers. Relative to XPA control littermates, no proliferative defects were observed in either cell type (Figure 2D and 2E). We further tested erythroblasts from double mutant CSB/XPA as well as single mutant CSB and XPA fetal livers and found no genotype-specific proliferative differences (Figure 2F).

Finally, we tested hypersensitivity of MEFs to acute oxidative stress induced by ionizing radiation, paraquat, and tert-butyl hydroperoxide. Similar to *Xpg^−/−^* MEFs [24,27], we found no significant differences between any of the genotypes (Figure 2G and unpublished data). As a control, all single and double homozygous mutants tested were hypersensitive to UV-C, consistent with their NER deficiencies (Figure 2G and unpublished data). In conclusion, it is unlikely that premature senescence or cell death observed in proliferating primary cell cultures under conditions of chronic or acute oxidative stress are related to the symptoms common amongst the various progeroid syndromes listed in Table 1.

### Purkinje Cell Death Is a Late Event in Disease Progression

**Figure 3:**
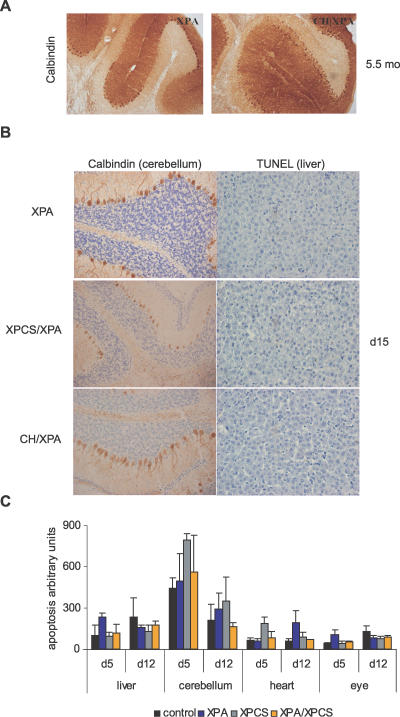
Proliferation and Cell Death Do Not Correlate with Onset or Severity of Progeroid NER Syndrome (A) Anti-calbindin staining (brown) of Purkinje neurons of 5.5-mo-old CH/XPA and control XPA mice (magnification 100×). (B) TUNEL staining (brown) with hematoxylin costain (blue) of liver sections and anti-calbindin immunostaining (brown) with hematoxylin costain (blue) of cerebellar sections of representative XPCS/XPA, CH/XPA, and control XPA mice at 15 d of age (magnification 400×). (C) Ligation-mediated PCR-based analysis of apoptosis in liver, cerebellum, heart, and eye of postnatal day 5 (d5) and day 12 (d12) control (*n* = 2–3), XPA (*n* = 3), XPCS (*n* = 3), and XPA/XPCS (*n* = 3–4) mice. The relative amount of laddered PCR products representing apoptosis is presented in arbitrary units on the *y*-axis; error bars indicate SEM.

We next looked for differences in cell death or proliferation in vivo using one of the severe progeroid NER syndromes, XPCS/XPA, in comparison to single mutant (XPCS and XPA) and heterozygous (control) littermates at postnatal days 5, 12, and 15 coincident with growth retardation and ataxia but prior to signs of weight loss or morbidity. We focused on this double homozygous mutant because of the 100% penetrance of mortality around weaning (Table 1). We first examined cerebellar Purkinje neurons, one of the few cell types consistently affected in severe progeroid NER syndromes as reported for XPG, CSB/XPA, and XPCS/XPA (Table 1). Despite disturbed gait and balance problems consistent with cerebellar ataxia, CH/XPA mice showed no detectable degeneration or loss of Purkinje neurons or abnormalities in cerebellar morphology upon sacrifice at the age of 5.5 mo (Figure 3A). Similarly, Purkinje neurons were morphologically intact in all mutant and control XPD/XPA animals at postnatal day 15 (Figure 3B). These results were unexpected based on the almost complete loss of Purkinje cells by postnatal day 20 in XPCS/XPA mice [9] as well as XPG mice [28].

Except for cellular loss in the cerebellum very late in disease progression, gross pathological analysis of double mutant XPCS/XPA mice did not reveal overt defects in any other major organ system investigated, similar to reports in TTD/XPA and CSB/XPA mice [20,21]. Furthermore, we failed to observe polyploidization of liver nuclei (Figure 3B) as in *Ercc1^−/−^* and *Xpf^−/−^*mice [25,29,30]. Cellular proliferation, as measured by BrdU incorporation and Ki67 and PCNA immunohistochemistry, in 5- and 12-d-old mice did not reveal evident genotype-specific differences in the number of replicating cells in the intestine, liver, kidney, heart, or lung (unpublished data). Nor did we find evidence of increased cell death/apoptosis in hematoxylin-and-eosin- or TUNEL-stained sections of various organs or following sensitive ligation-mediated PCR-based analysis of DNA from organs including liver, cerebellum, heart, and eye (Figure 3C and unpublished data). Taken together, these in vitro and in vivo data support neither a vicious cycle of cell death and proliferative exhaustion nor a general cell-autonomous proliferative defect per se as causative of the cachectic dwarfism and failure to thrive in the developmental period that precedes severe pathology and death before weaning in most progeroid NER mice.

### Symptoms of Progeroid NER Syndrome Resemble Those of Long-Lived Dwarfism and Calorie Restriction

**Figure 4:**
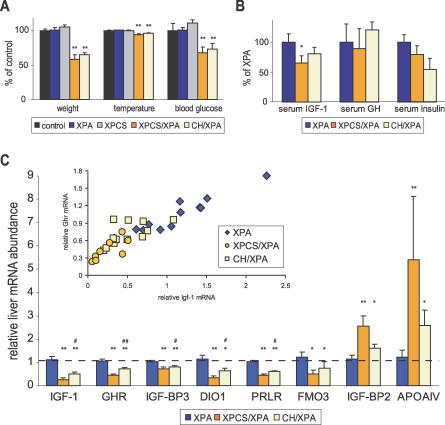
Perturbation of GH/IGF-1 Axis Correlates with Severity of NER Progeria (A) Body weight, temperature, and whole blood glucose of anesthetized WT control (*n* = 4, black); single mutant XPA (*n* = 23, blue) and XPCS (*n* = 6, grey); and double mutant XPCS/XPA (*n* = 6, orange) and CH/XPA (*n* = 13, beige) animals relative to WT controls. Asterisks indicate statistical significance versus WT controls (**, *p* < 0.01); error bars indicate SEM. (B) Serum IGF-1, GH, and insulin as determined by ELISA in XPCS/XPA (*n* = 5) and CH/XPA (*n* = 7) relative to XPA (*n* = 6) animals. Asterisk indicates statistical significance versus XPA controls (*, *p* < 0.05); error bars indicate SEM. (C) Abundance of liver mRNAs encoding IGF-1, GHR, IGF-BP3, DIO1, prolactin receptor (PRLR), FMO3, IGF-BP2, and APOAIV in XPCS/XPA (*n* = 8) and CH/XPA (*n* = 12) relative to XPA (*n* = 12) mice as determined by quantitative real-time PCR. Asterisks indicate statistical significance versus XPA controls (*, *p* < 0.05; **, *p* < 0.01); pound signs indicate statistical significance between XPCS/XPA and CH/XPA (#, *p* < 0.05; ##, *p* < 0.01); error bars indicate SEM. Inset: correlation between relative IGF-1 and GHR mRNA expression values in individual animals; correlation coefficients per group: XPA, 0.96 (blue diamonds); XPCS/XPA, 0.76 (orange circles); CH/XPA, 0.84 (yellow squares); overall, 0.92.

We next looked for parameters that were partially rescued by *Xpd* compound heterozygosity in CH/XPA mice compared to XPCS/XPA mice in order to elucidate those parameters that are important in disease etiology. We focused initially on metabolic parameters including body weight, temperature, and blood glucose in anesthetized mice. As shown in Figure 4A, significant differences between mutants and controls were observed in body weight, temperature, and blood glucose in anesthetized mice upon sacrifice at postnatal day 15. Interestingly, however, these differences did not reach statistical significance between XPCS/XPA and CH/XPA animals despite the large difference in lifespan.

Growth retardation, hypothermia, hypoglycemia (Figure 4A), and reduced body mass despite normal to increased food intake per gram body weight (Figure 1B) are phenotypes shared by long-lived mice with defects in growth hormone (GH) signaling, including GH-deficient Ames and Snell dwarfs and GH-resistant GH receptor (GHR) knock-out (GHR-KO) mice [31,32]. GH mediates its effects largely through the increased secretion of insulin-like growth factor 1 (IGF-1) in target tissues including the liver. To determine the status of the GH/IGF-1 axis in progeroid NER syndrome, we measured total IGF-1 in serum of postnatal day 15 animals (Figure 4B). Compared to normally sized XPA animals, serum IGF-1 was reduced in both XPCS/XPA and CH/XPA pups, although only to a level of statistical significance in XPCS/XPA animals (*p* < 0.05). IGF-1 mRNA from liver, the major source of circulating IGF-1, was significantly reduced in both (Figure 4C, *p* < 0.05). As a control, relative abundance of IGF-1 and a number of other mRNAs in single mutant XPA, XPCS, and TTD livers was not significantly different from heterozygous controls (unpublished data).

We next measured GH in the serum (Figure 4B). Despite considerable interanimal variation, likely due to the pulsatile nature of GH secretion from the pituitary [33], levels were in the range of control XPA animals independent of genotype. Thus, dwarfism in NER progeria is not likely a primary pituitary defect as in Ames and Snell dwarf mice, in which the anterior pituitary fails to develop properly [34]. Consistent with this, no histological or functional differences were detected in the pituitary gland of another progeroid NER mutant, CSB/XPA [35]. GH insensitivity, as in GHR-KO mice, can also cause dwarfism [36,37] even in the presence of excess GH. Indeed, liver GHR mRNA abundance was significantly reduced in both XPCS/XPA and CH/XPA pups. Despite a range of expression levels, maintenance of the strong correlation between GHR and IGF-1 mRNA in individual CH/XPA (*r* = 0.84, *n* = 12) and XPCS/XPA (*r* = 0.76, *n* = 8) animals relative to control XPA animals (*r* = 0.96, *n* = 12) suggests that coordinated transcriptional regulation is intact in progeroid NER syndrome (Figure 4C, inset) and that GHR mRNA levels are a relevant indicator of GHR protein function.

Calorie restriction and GH deficiency/insensitivity both result in hypoglycemia and hypoinsulinemia. Increased sensitivity to the effects of insulin is thought to underlie the low blood sugar and may also be a key to extended longevity [31]. We measured serum insulin from day 15 animals and found, similar to dwarves and calorie-restricted animals, no evidence of hyperinsulinemia as a cause of hypoglycemia (Figure 4B). IGF-1, IGF-1 binding protein 3 (IGF-BP3, also known as GH-dependent binding protein), and acid-labile subunit (ALS) form a ternary complex in the serum and are all reduced in GH insensitivity disorders in proportion to the severity of GH resistance [38]. In XPCS/XPA and CH/XPA animals, liver IGF-BP3 and ALS mRNA levels were reduced in addition to that of IGF-1, although the reduction in ALS expression in CH/XPA did not meet the criteria for statistical significance (Figure 4C and unpublished data). Other key genes involved in postnatal growth via the somatotroph axis, including deiodinase 1 (DIO1), which activates thyroid hormone precursor, and the prolactin hormone receptor, were significantly reduced as well (Figure 4C). Within this signature group of five somatotroph axis genes, mRNA expression levels were significantly different not only between NER progeroid animals and controls, but also between XPCS/XPA and CH/XPA animals; in each case, downregulation was more severe in XPCS/XPA animals. Thus, on the level of gene expression, downregulation of the somatotropic GH/IGF-1 axis correlated with the severity of NER progeria.

IGF-1, ALS, and prolactin receptor are three of a surprisingly limited number of liver transcripts reported to be altered in the long-lived GHR-KO dwarf (only ten genes at a significance of *p* < 0.001), while in Snell dwarf mice and calorie-restricted mice, hundreds of liver transcripts are differentially expressed using the same experimental platform and statistical criteria [39]. From this list of ten transcripts, we additionally tested IGF-BP2, flavin-containing monoxygenase 3 (FMO3), and apolipoprotein A-IV (APOAIV) expression. Circulating concentrations of IGF-BP2 are known to be inversely related to GH status [38] and were significantly upregulated in progeroid NER mice, further underlining the downregulation of the somatotropic axis. FMO3, which is involved in the oxidative breakdown of xenobiotic compounds and downregulated in GHR-KO mice, was also downregulated in progeroid NER mice. Finally, APOAIV mRNA, encoding a protein involved in serum lipid transport that is ∼10-fold reduced in GHR-KO mice but 4- to 5-fold increased upon 24–48 h of starvation [40], was 3- to 6-fold increased in NER-deficient mice. In conclusion, many but not all characteristics of both CH/XPA and XPCS/XPA mice resembled those of either calorie restriction or genetic dwarfism.

### An Adaptive Response to Stress in Progeroid NER Syndrome

**Figure 5:**
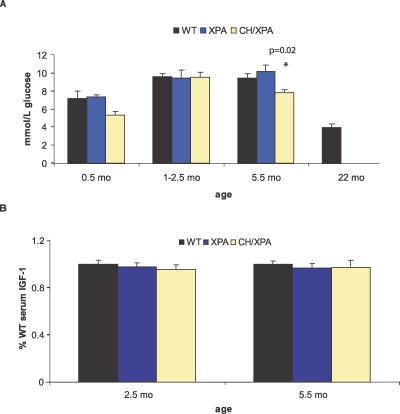
Kinetics of Blood Glucose and Serum IGF-1 Levels throughout the Life of CH/XPA Mice Longitudinal measurements of (A) blood glucose and (B) serum IGF-1 in CH/XPA versus XPA and WT control mice. Error bars indicate SEM; 5–15 animals were used per genotype per timepoint. *p-*Value indicates the significance of the difference relative to WT at that timepoint.

In genetic dwarfism, differences in serum IGF-1 and glucose metabolism are caused by congenital, irreversible defects in components of the postnatal growth axis itself. In calorie-restricted animals, these differences are reversible and represent an adaptive response to the stress of nutritional deprivation. We followed blood glucose and serum IGF-1 levels in CH/XPA mice over their lifespan to see if the phenotypic overlap with dwarf and calorie-restricted animals was more a constitutive defect or an adaptive response. At 10 wk of age, blood glucose and serum IGF-1 levels were normal in CH/XPA animals relative to WT and XPA controls (Figure 5). This strongly suggests that the reduction of these components during early postnatal development (Figure 4) represents an adaptive response rather than a constitutive defect. At the end of their lives, IGF-1 levels remained within the normal range, while blood glucose levels once again fell significantly below control WT and XPA levels. Low serum glucose levels were also observed in the long-term survivors of a cohort of WT mice at the end of their lives (approximately 2 y; Figure 5).

### Is Somatotroph Axis Dampening Common to Progeroid Genome Instability Disorders?

**Figure 6:**
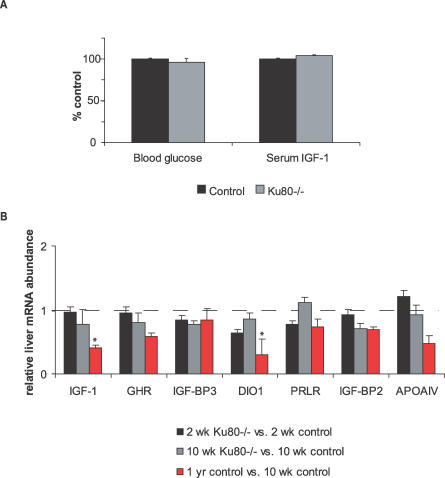
Analysis of Blood Glucose, Serum IGF-1, and Liver Gene Expression Profiling in NHEJ-Deficient *Ku80^−/−^* Mice (A) Glucose and IGF-1 levels in the blood serum of 2-wk-old *Ku80^−/−^* versus WT or heterozygous littermate controls. Error bars indicate SEM; six animals were used per group. (B) Abundance of liver mRNAs encoding IGF-1, GHR, IGF-BP3, DIO1, prolactin receptor (PRLR), FMO3, IGF-BP2, and APOAIV) in 2-wk-old *Ku80^−/−^* (*n* = 6) versus 2-wk-old WT or heterozygous littermate controls (*n* = 6); and both 10-wk-old *Ku80^−/−^* (*n* = 4) and ∼1-y-old versus 10-wk-old controls (*n* = 5) as determined by quantitative real-time PCR. Error bars indicate SEM between experiments.

Cachectic dwarfism is common in mouse models of genome instability with segmental progeria, and has been attributed in many cases to cell-autonomous proliferative defects (premature senescence) as observed in cultured MEFs [3]. We next asked whether the dampening of the somatotroph axis observed here in progeroid NER disorder is also found in cachectic dwarfism caused by a different primary DNA repair defect. *Ku80^−/−^* mice, which are defective in the repair of double-strand DNA breaks via the nonhomologous endjoining (NHEJ) pathway, are smaller than control littermates from embryonic day 17.5 on [41] and display multiple symptoms of premature senescence including kyphosis, atrophic skin, hepatocellular degeneration, and reduced lifespan due to cancer and sepsis [42]. We looked at blood glucose and serum IGF-1 levels in 2-wk-old animals and found no differences between *Ku80^−/−^* and WT or heterozygous littermate control mice (Figure 6A). Next, we analyzed liver mRNA expression of signature somatotroph axis genes in *Ku80^−/−^* mice versus littermate controls using quantitative real-time PCR, the method found above to be most sensitive at identifying subtle differences between progeroid NER mice. Despite having 50%–60% reduced weight relative to littermate controls, *Ku80^−/−^* mice did not display evidence of somatotropic axis dampening as observed in progeroid NER mice of the same age (Figure 6B). Nor was involvement of long-lived dwarf GHR-KO signature genes such as *ApoAIV* detected. We also found no evidence of somatotroph dampening in *Ku80^−/−^* mice at 10 wk of age, when the activity of this axis should be near its peak. As a control for the ability to detect somatotropic axis dampening in 10-wk-old mice, we compared them to older animals (∼1 y of age) from the same animal facility and observed the expected age-related reduction in transcript abundance of somatotroph axis genes including those encoding IGF-1 and DIO1. In conclusion, unlike in NER progeria, we found no evidence of reduced GH/IGF-1 signaling contributing to cachectic dwarfism in *Ku80^−/−^* mice.

## Discussion

We describe a new mouse model of progeroid NER syndrome created by combining two different mutant *Xpd* alleles in an *Xpa^−/−^* background. A major benefit of this model over existing models (Table 1) is its ability to survive weaning with high penetrance and its having a lifespan proportional to relevant progeroid NER disorders including CS. Furthermore, it is simpler than XPG, XPF, and ERCC1 models that additionally have profound cell-autonomous proliferative defects. Using this model we found evidence for an adaptive response to genome instability during postnatal development involving dampening of the somatotropic GH/IGF-1 axis and perturbation of glucose homeostasis. This response appears so far specific to excision repair disorders, as it was not observed in NHEJ-deficient mice despite cachectic dwarfism and several progeroid features.

### Uncoupling of Dwarfism and Progeria from Premature Fibroblast Senescence in Progeroid NER Syndrome

There is a strong correlation between genomic instability disorders with symptoms of progeria and premature senescence of the corresponding cultured fibroblasts, for example, in Werner syndrome, Hutchinson-Gilford progeria, and ataxia telangiectasia in humans [43], and BUB1, PASG, DNA-PK, ERCC1, and KU80 mutants in mouse [3]. As a result, cell-autonomous proliferative defects are generally regarded as strong candidates underlying progeroid phenotypes, including cachectic dwarfism [3,41]. Mechanistically, this may be due to not only reduced proliferative capacity per se but also altered characteristics of senescent cells such as secretion of inflammatory cytokines and extracellular matrix-degrading enzymes [44]. Here, in related NER progerias (CH/XPA, XPCS/XPA, TTD/XPA, and CSB/XPA), we demonstrated that cell-autonomous proliferative defects as observed in two different primary cell types, fibroblasts and erythroblasts, are not an absolute prerequisite for postnatal growth retardation or progeria. This makes these NER-associated progerias exceptional amongst mouse progeroid syndromes caused by genome instability. Consistent with these findings in mice, CS and TTD are peculiar amongst human progerias not only in their lack of cancer predisposition but also in the failure of cells to undergo premature senescence in culture [43]. Taken together, these data suggest that defects particularly in transcription-coupled NER are not growth limiting in proliferating cells. This may be due to their ability to repair (or tolerate) DNA damage by a variety of replication-dependent, NER-independent mechanisms. In contrast, nondividing postmitotic cells such as neurons may be at greater risk for the effects of transcription-coupled NER deficiencies precisely because they do not have the opportunity to “clean” damaged genes or dilute the damage by DNA replication. At the same time, neurons appear to downregulate their ability to repair lesions via the global genome NER pathway [45].

Instead of revealing any cell-autonomous proliferative defect, our data demonstrate dampening of the postnatal GH/IGF-1 axis, making this a much more likely explanation underlying reduced postnatal growth and small size. The inability to detect gross differences in cell death or proliferation in vivo except in specific cell types (i.e., cerebellar granular neurons [20]) or at the end stage of disease (i.e., cerebellar Purkinje loss [9]) does not rule out the contribution of small but significant differences in the number of proliferating or dying cells due to a lack of growth and survival signals. Such differences are likely to be found in specific cell compartments, such as slowly cycling (label-retaining) cells of the germinal layer or chondrocytes in the proliferative zone of the growth plate responsible for longitudinal bone growth, which are stimulated by GH and IGF-1, respectively [46].

### Perturbation of Somatotropic GH/IGF-1 Axis and Glucose Homeostasis: Constitutive Defect or Adaptive Response?

We observed a number of similarities of progeroid NER mice with long-lived dwarf and calorie-restricted mice, including small size, hypoglycemia, and reduced serum IGF-1, indicative of dampening of the postnatal GH/IGF-1 growth axis. In a number of model organisms including worms and mice, suppression of the GH/IGF-1 axis is associated with increased stress resistance and extended longevity [31]. Reduced serum IGF-1 can be caused by genetic defects in structural components of the somatotropic axis itself (Ames and Snell dwarfs and GHR-KO mice) or by an adaptive response to environmental cues such as starvation or calorie restriction. For example, Ames and Snell dwarf mice suffer congenital hypopituitarism and lack a number of pituitary hormones including GH from birth. Upon dietary restriction, the only intervention known to extend lifespan in mammals, reduced blood glucose and IGF-1 appear to be key components of an adaptive response to stress [47,48] and are entirely reversible. The apparent logic behind this reversible response is to shift the metabolism away from energy consumption directed at growth or reproduction toward energy conservation and somatic maintenance with a concomitant increase in the ability of cells to cope with stress, with an overall aim of enhancing survival until favorable conditions return.

In CH/XPA mice, blood glucose and serum IGF-1 were reduced relative to controls at 2 wk of age but returned to normal by 10 wk of age. These data strongly argue against any structural defect in the components of the somatotropic axis or glucose homeostasis itself. This also makes what is happening in CH/XPA mice potentially quite different from what has been proposed for progeroid SIRT6^−/−^ mice. Based on these animals' low blood glucose and serum IGF-1, failure to thrive, and death with high penetrance before weaning [49], the authors proposed an important role for SIRT6 in maintaining organismal homeostasis, in addition to a role in DNA repair via the base excision repair (BER) pathway. In CH/XPA mice, we believe that the reduced blood sugar and serum IGF-1 during early postnatal development are components of an adaptive response to some form of stress experienced during this period before weaning. Although the nature of this stress is unknown, it appears to be initiated soon after birth and may involve the dramatic transition from prenatal to postnatal environment, including a different oxygen concentration and a switch in energy acquisition source from the mother's blood via her bloodstream to the mother's milk via the pup's own digestive system, combined with a period of rapid growth.

Interestingly, although serum IGF-1 levels remained in the normal range at least some time before the death of the CH/XPA animals, blood glucose levels dropped significantly below those of age-matched controls, indicating that low IGF-1 levels do not always precede low glucose. We also observed reduced glucose levels in 15 of the oldest survivors of a longevity cohort of WT mice. Recently it was reported that senescent terminal weight loss in rats correlated with extended longevity [50]. It is therefore tempting to speculate that reduced size and decreased blood glucose are components of an adaptive response intended to extend longevity in both normal and some forms of pathological aging rather than the unintended consequences of random degenerative processes.

Differences between progeroid NER mice and long-lived dwarf mice, on the other hand, may be better explained by defects in the transcription function of the transcription factor II H complex to which XPD belongs. For example, altered fat metabolism in CH/XPA mice may be related to reported defects in the activation of peroxisome proliferator-activated receptors implicated in lipid metabolism [51].

### Paradox of IGF-1 Reduction: Extended Versus Shortened Lifespan

**Figure 7:**
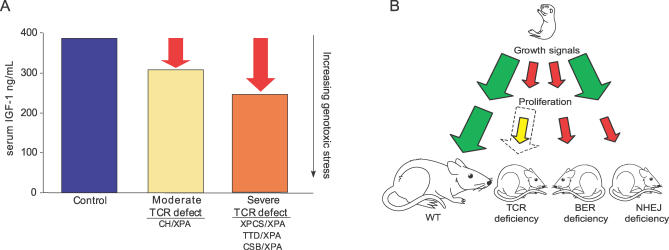
Model for GH/IGF-1 Axis and Cell Proliferation Involvement in Segmental Progeroid Genome Instability Disorders (A) Inverse correlation between IGF-1 and lifespan in progeroid NER syndrome. Decreased IGF-1 signaling usually correlates positively with increased longevity; in XPD/XPA mutants lower serum IGF-1 (*y*-axis on the left) instead correlates inversely with lifespan. To explain this apparent paradox, we interpret serum IGF-1 levels as indicative of the magnitude of the perceived genotoxic stress (red arrows, *y*-axis on the right). This genotoxic stress may overrule the efficacy of reduced IGF-1 in XPCS/XPA and TTD/XPA animals, while in CH/XPA animals compound heterozygosity partially complements the defect, relieving genotoxic stress and reducing the corresponding stress response. (B) Model for the differential contribution of cell-autonomous proliferative defects and systemic growth axis perturbation to progeria in genome instability disorders. Growth signals from the GH/IGF-1 somatotropic axis are dampened in progeroid NER syndrome (small red arrow), leading to postnatal growth deficiency; cells removed from this environment and provided adequate growth signals proliferate normally (dotted arrow represents WT proliferative capacity). In other segmental progerias, such as SIRT6 deficiency with a reported defect in BER, both appear to be affected. Alternately, cellular proliferation can be affected without apparent involvement of the somatotropic axis as in NHEJ-deficient *Ku80^−/−^* mice.

Although reduced serum IGF-1 on its own does not result in dwarfism or extended longevity in mice (liver IGF-1 knock-out; [52]), it correlates with both in most reported cases. Comparative liver transcriptome analyses of long-lived mice with dwarfism versus calorie restriction also point to IGF-1 as one of the few consistent predictors of extended longevity [39]. If reduced IGF-1 mRNA correlates with increased longevity, why were liver IGF-1 mRNA levels observed here lowest in XPCS/XPA animals with the shortest lifespan and intermediate in CH/XPA mice with intermediate longevity? Our data demonstrate that IGF-1 dampening is reversible (at least in the CH/XPA mice) and thus most likely an adaptive response to genotoxic stress. When considering the benefits of reduced IGF-1, one must also in this case consider the magnitude of the stress that triggers this response. At some level of damage, this stress may overrule the efficacy of the response even at its maximal beneficial level. According to this interpretation, the magnitude of the dampening of the postnatal growth axis does not correlate directly to lifespan extension, but to the magnitude of the perceived stress, be it starvation or genome instability, and is independent of whether that stress response is successful in extending longevity (Figure 7A). Thus in XPCS/XPA and TTD/XPA mice, this results in death before weaning with high penetrance despite the maximal stress response; compound heterozygosity at the *Xpd* locus, which partially complements this defect, results in a less robust stress response because of less or slower damage accumulation, resulting in longer lifespan. Whether or not this response is adaptive or maladaptive and what actually triggers it (i.e., central nervous system dysfunction or malnutrition) remain important questions for future research.

### Uncoupling of Postnatal Growth Axis Dampening from Dwarfism in Progeroid Genome Instability Disorders

In contrast to certain progeroid NER disorders, cultured *Ku80^−/−^* fibroblasts display cell-autonomous proliferative defects [26]. Furthermore, *Ku80^−/−^* dwarfism is not exclusively postnatal (animals are already smaller at birth [41]), and we found no evidence of perturbation of the GH/IGF-1 postnatal growth axis at 2 wk, as in progeroid NER disorder, or at 10 wk, near the peak of somatotroph axis activity. This suggests a model in which cachectic dwarfism and progeria can be caused by at least two fundamentally distinct mechanisms: perturbation of postnatal growth signaling independent of cell-autonomous proliferative defects, as in NER progeria, or cell-autonomous proliferative defects without perturbation of the somatotroph axis, as in KU80 deficiency. Because these two scenarios are not necessarily mutually exclusive (for example, *Ercc1^−/−^* and *Xpg^−/−^* mice display symptoms of NER progeria [Table 1] but also have cell-autonomous proliferative defects; SIRT6^−/−^ mice with a reported BER deficiency also display cell-autonomous proliferative defects in addition to reduced serum IGF-1), dwarfism can likely result from combinations of both. In Figure 7B, we summarize how defects in growth factor environment and cell-autonomous proliferative capacity may independently or together produce the symptoms of segmental progeria due to NER dysfunction or other defects in genome maintenance.

### Conclusions

Taken together, these data reveal perturbation of the GH/IGF-1 axis as a novel mechanism underlying the common symptoms of progeroid NER syndromes. Downregulation of IGF-1 may thus be part of a general stress response activated not only upon conditions such as food scarcity but also in response to the accumulation of certain types of DNA damage via congenital repair defects and possibly natural aging. In support of this interpretation, end-of-life pathology in TTD mice is consistent with both segmental accelerated aging (e.g., increased lipofuscin accumulation in the liver) and calorie restriction (e.g., reduced incidence of pituitary adenomas) [53]; TTD liver gene expression also resembled that of long-lived dwarf mice (Y. Suh, J. Y. Park, M. O. Cho, S. Leonard, B. Calder , unpublished data). Similarly, transcriptome analysis of CSB/XPA livers revealed significant downregulation of the somatotroph axis as well as upregulation of antioxidant defense [35]. Finally, reduced serum IGF-1 and glucose levels were observed in segmental progeroid SIRT6 knock-out mice, with a tentative link to a defect in DNA repair via the BER pathway [49]. The relevance of these findings from mouse models to the related human disorders remains to be determined. Downregulation of neuroendocrine GH/IGF-1 signaling has not been a consistent finding in CS or TTD patients, although the relative paucity of both patients and data prevents firm conclusions from being drawn [16–18]. However, as evidenced by the reduced cancer incidence in hypopituitary dwarf mice [31], it is a plausible mechanism contributing to the lack of cancer observed in these individuals [16–18].

## Materials and Methods

### Mice.

*Xpa^−/−^, Xpd^TTD^* (XPD^R722W^) and *Xpd^XPCS^* (XPD^G602D^) mice were generated as described previously [9,10,23]. Mice were in a mixed 129Ola|C57BL/6|FVB background. All experiments involving mice were evaluated and approved by the National Committee for Genetic Identification of Organisms and the Animal Ethics Committee and were conducted according to national and international guidelines.

### Cells.

Embryonic fibroblasts from day 13.5 embryos were dispersed by mechanical disruption with an Ultra-Turrax T25 Basic (Ika, http://www.ika.net) and cultured in a 50:50 mix of DMEM:Ham's F10 supplemented with 10% fetal bovine serum and antibiotics (pen/strep) in a mixed gas incubator with 5% CO_2_ and 3% O_2_ (passage 0) unless otherwise noted. For long-term proliferation assays, cells were counted using a Coulter Multisizer Z2 (Beckman Coulter, http://www.beckmancoulter.com) , seeded at 375,000 cells per 10-cm dish, and cultured at both 3% and atmospheric oxygen tensions. For subsequent passages, cells were trypsinized before they reached ∼80% confluence. For acute stress sensitivity assays, early passage cells (passage 1–5) were plated in duplo in six-well plates at a density of 20,000 cells per well and treated with the appropriate agent the following day. Cells were counted 4–5 d after treatment using the Multisizer Z2 and plotted as a percentage of the total number of cells from the corresponding untreated cell line. Dermal fibroblasts were prepared from the isolated tail skin of euthanized animals. Following mincing with a razor, tail skin was incubated overnight in a mixed gas incubator with 5% CO_2_ and 3% O_2_ in DMEM supplemented with 20% fetal bovine serum and antibiotics and 40 mg/ml Type II Collagenase (Invitrogen, http://www.invitrogen.com). The next day, cells were resuspended, passaged through a 40-μm filter, washed, and replated in medium without collagenase. Long-term proliferation assays were performed as above except that cells were plated at a slightly higher density (500,000 cells per 10-cm round bottom dish). Erythroblast cultures were derived from fetal livers removed from day 13.5 embryos. Progenitors were cultured as previously described [54] under normoxic conditions.

### Apoptosis.

Apoptosis was measured by ligation-mediated PCR using the Apo-Alert LM-PCR Ladder Assay Kit in combination with Advantage cDNA Polymerase mix (both from Clontech, http://www.clontech.com) according to the manufacturer's instructions. TUNEL assays were performed using the ApoTag Plus Peroxidase In Situ Apoptosis Detection Kit (S7101, Chemicon International, http://www.chemicon.com) according to the manufacturer's instructions.

### Immunohistochemistry.

For histological examination, mouse samples fixed in formalin were embedded in paraffin, sectioned, rehydrated, and stained with hematoxylin and eosin or monoclonal antibodies against PCNA, Ki67, or calbindin and visualized based on HRP-conjugated secondary antibodies. TUNEL staining was performed using ApopTag Plus Peroxidase In Situ Apoptosis Detection Kit (Chemicon International) according to the manufacturer's instructions.

### Physiologic parameters.

Food intake was measured by weighing remaining dry food given to individually housed animals (three per genotype) and the corresponding animals themselves each day over the period of 4 d; the experiment was repeated twice with different litters at different times. Body temperature was measured in animals immediately following anesthetization with Isofluran (Baxter, http://www.baxter.com) by inserting a surgical thermometer into a small hole cut in the abdominal cavity or into the rectum. Blood was collected following decapitation. Glucose was measured in whole blood using the Freestyle Mini blood glucose meter (Abbott Laboratories, http://www.abbott.com) with a range of detection between 1.1 and 27.8 mmol/l. Serum was prepared by low speed centrifugation following clotting. Serum IGF-1, GH, and insulin were measured by ELISA using Mouse/Rat IGF-I (DSL-10–2900), with a range of detection between 50 and 3,500 ng/ml, and Mouse/Rat GH (DSL-10–72100) with a range of detection between 1 and 100 ng/ml, both from Diagnostic Systems Laboratories (http://www.dslabs.com), and Ultrasensitive Mouse Insulin ELISA (10-1150-01), with a range of detection between 0.188 and 3.75 μg/l, from Mercodia (http://www.mercodia.com), according to the manufacturer's instructions.

### Quantitative real-time PCR.

Total RNA was extracted from the liver using TRIzol reagent (Invitrogen) and oligodT or hexamer-primed cDNA synthesized using SuperScript II (Invitrogen) according to the manufacturer's instructions. Quantitative real-time PCR was performed using an Opticon2 DNA Engine and a MyIQ (Bio-Rad, http://www.bio-rad.com) with SYBR Green incorporation. Relative expression was calculated using the equation 1.8^−(ΔCt sample – ΔCt control)^ [55]. Each sample was tested in duplo at least two times.

## Supporting Information

Video S1 Progeroid Features of CH/XPA Mice CH/XPA mice are shown relative to littermate controls at 2 mo of age displaying unusual daytime eating behavior and abnormal clasping of hind limbs in a tail-hanging test; and again at 5 mo of age displaying disturbed gait, loss of balance, and kyphosis.
(3.0 MB WMV)

## Abbreviations

ALS: acid-labile subunit
APOAIV: apolipoprotein A-IV
BER: base excision repair
CH/XPA: compound heterozygous *Xpd^XPCS/TTD^/Xpa^−/−^*
CS: Cockayne syndrome
DIO1: deiodinase 1
FMO3: flavin-containing monoxygenase 3
GH: growth hormone
GHR: growth hormone receptor
GHR-KO: growth hormone receptor knock-out
IGF-1: insulin-like growth factor 1
IGF-BP: IGF-1 binding protein
MEF: mouse embryonic fibroblast
NER: nucleotide excision repair
NHEJ: nonhomologous endjoining
SEM: standard error of the mean
TTD: trichothiodystrophy
XP: xeroderma pigmentosum
XPCS: xeroderma pigmentosum combined with Cockayne syndrome
WT: wild-type
