# Correction: Adaptive Stress Response in Segmental Progeria Resembles Long-Lived Dwarfism and Calorie Restriction in Mice

**DOI:** 10.1371/annotation/31502636-9bdf-4cb1-b995-4a30a607e316

**Published:** 2008-10-17

**Authors:** Marieke van de Ven, Jaan-Olle Andressoo, Valerie B Holcomb, Marieke von Lindern, Willeke M. C Jong, Chris I. De Zeeuw, Yousin Suh, Paul Hasty, Jan H. J Hoeijmakers, Gijsbertus T. J van der Horst, James R Mitchell

J. Hoeijmakers wishes to declare the following competing interest, which should have been indicated at the time of publication. Dr. Hoeijmakers owned shares in DNage, a company set up by Erasmus MC, and subsequently acquired by Pharming. However, for clarification, no materials or support were received from either company, and no agreements were in place concerning the execution or publication of this work.

